# rPCMP: robust *p*-value combination by multiple partitions with applications to ATAC-seq data

**DOI:** 10.1186/s12918-018-0661-z

**Published:** 2018-12-31

**Authors:** Menglan Cai, Limin Li

**Affiliations:** School of Mathematics and Statistics, Xi’an Jiaotong University, Xianning West 28, Xi’an, China

**Keywords:** group *p*-value, multiple partitions, ATAC-seq

## Abstract

**Background:**

Evaluating the significance for a group of genes or proteins in a pathway or biological process for a disease could help researchers understand the mechanism of the disease. For example, identifying related pathways or gene functions for chromatin states of tumor-specific T cells will help determine whether T cells could reprogram or not, and further help design the cancer treatment strategy. Some existing *p*-value combination methods can be used in this scenario. However, these methods suffer from different disadvantages, and thus it is still challenging to design more powerful and robust statistical method.

**Results:**

The existing method of Group combined *p*-value (GCP) first partitions *p*-values to several groups using a set of several truncation points, but the method is often sensitive to these truncation points. Another method of adaptive rank truncated product method(ARTP) makes use of multiple truncation integers to adaptively combine the smallest *p*-values, but the method loses statistical power since it ignores the larger *p*-values. To tackle these problems, we propose a robust *p*-value combination method (rPCMP) by considering multiple partitions of *p*-values with different sets of truncation points. The proposed rPCMP statistic have a three-layer hierarchical structure. The inner-layer considers a statistic which combines *p*-values in a specified interval defined by two thresholds points, the intermediate-layer uses a GCP statistic which optimizes the statistic from the inner layer for a partition set of threshold points, and the outer-layer integrates the GCP statistic from multiple partitions of *p*-values. The empirical distribution of statistic under null distribution could be estimated by permutation procedure.

**Conclusions:**

Our proposed rPCMP method has been shown to be more robust and have higher statistical power. Simulation study shows that our method can effectively control the type I error rates and have higher statistical power than the existing methods. We finally apply our rPCMP method to an ATAC-seq dataset for discovering the related gene functions with chromatin states in mouse tumors T cell.

## Background

Genetic association analysis has been widely used to identify many associated genes with human complex diseases [1, 2]. In recent decades, the advances on biological techniques have made it possible to collect massive amounts of high-throughput datasets such as SNP data and gene expression data, which are often high dimensional, and have a large number of variables and a relatively small number of samples. A typical problem is to find out single nucleotide polymorphisms (SNPs) or genes related to corresponding diseases. Single-marker analysis could be done by two-sample test on each variable such as Fisher’s exact test, or Chi-squared test for categorical datasets, and two-sample t-test or Wilcoxon test for numerical datasets. However, a major disadvantage of single-marker based methods is that they do not consider the joint effects of multiple genetic variants which may have weak or moderate signals individually. The joint use of information from multiple markers may be more effective to reveal association between a genomic region and a trait than single marker analysis. In this scenario, gene-based, gene-set-based and pathway-based association tests provides a more powerful way in addition to the more widely used single marker association analysis. For example, one may want to test using SNP datasets whether a gene, including several or many SNPs, is significantly associated with a trait, or want to test using high-throughput gene expression datasets whether a biological pathway, including several genes, is significantly associated with a trait.

One method to detect the association between a gene, which may hosts a lot of SNPs, or a biological pathway, which may have many genes, and human complex diseases in large scale genetic studies is using the framework of logistic regression to learn the odd ratios of SNPs. However, this may not work due to the high-dimensional problem. Especially when the SNPs are in high linkage disequilibrium, the solution is not stable. Gene set enrichment analysis algorithm [3] has been proposed for the identification of disease related pathways by measuring the overrepresentation of disease-gene associations within a given pathway compared to a list of reference genes. The underlying null hypothesis is that the set of genes in a given pathway has no enrichment of association signals compared to the rest. In contrast, in this manuscript, we focus on testing for the effect of a specific pathway/gene set without reference to any larger gene list. The underlying global null hypothesis is that there is no association of the disease with any of the genes in the given gene set. A more promising strategy is to use univariate test which constructs marginal test for each variable first and then combine the *p*-values together by *p*-value combination methods to accumulate marginal signals.

The earliest method to combine individual *p*-values is Fisher’s combined probability test (FCT) [4], which is popularly used in many applications [5–7] or taken as part of the statistic in other more complicated *p*-value combination methods such as [8–10]. FCT basically combines *m* independent *p*-values into a test statistic, which is proved to follow a chi-square distribution with 2*m* degrees of freedom under null hypothesis. When the *p*-values are not independent, empirical distribution is suggested to use, otherwise the type I error rates may be inflated [8]. However, FCT method may lose power when the number of individuals in the gene set is large, or most of the individuals are not significant. Zaykin et. al. [8] propose a truncated product method(TPM) which takes the product of only those *p*-values less than some specified cut-off value *ξ* and to evaluate the probability of such a product under the null hypothesis. 0.05 is usually adopt as the cut-off in practice. Different from [8], Dudbridge et al. [9] use an alternative strategy to take rank truncated product (RTP) of the *K* most significant *p*-values as the statistic for the testing. However, the two methods of TPM and RTP are both sensitive to the parameters *ξ* or *K*, and an inappropriate truncation point can have a detrimental effect on the power. In order to overcome this problem, especially when there are a large number of *p*-values to be combined, [11] proposed adaptive rank truncated product method (ARTP) to optimize the selection of the truncation point with a set of candidates. The defined statistic is the minimum empirical *p*-value observed at different truncation points.

An alternative method for testing the overall null hypothesis is tail strength (TS) method. This TS test statistic is a function of ordered *p*-values [12], which has an asymptotic normal distribution with mean of 0 and variance of 1/*m* under the null hypothesis if the *mp*-values are independent. Similar to [8, 13] defines a new truncated tail strength (TTS) statistic for testing the null hypothesis by removing *p*-values larger than a cutoff. The TTS statistic appears to have good properties, especially when there are a large number of independent tests in one dataset.

More recently, [14] adopts a sequential method for combining information from correlated *p*-values and presents the SEQ algorithm for correlated *p*-values. Hu et al. [10] defines a GCP statistic by using two functions log and the cumulative distribution function of two degrees of freedom to combine the *p*-values and show more power than FCT when these *p*-values are correlated and few *p*-values show significances. In GCP method, *p*-values are divided to several groups first by thresholds, and then constructed into a statistic with each group. However, when the number of individual tests is large, the performance of GCP is very sensitive to the selection of thresholds.

In this work, we propose a more robust statistical method called rPCMP to improve GCP method, by using multiple partitions of *p*-values. Borrowing the idea from ARTP, which takes several truncation points, our rPCMP takes several sets of thresholds to divide *p*-values to groups for several times. The defined rPCMP havs three-layer structure, which could be empirically estimated by a permutation procedure. Extensive simulations studies show that our proposed rPCAMP test method perform more powerful than some existing *p*-value combination methods, with low type I error rates. Our method is finally applied to a ATAC-seq dataset, to find the related gene functions for chromatin states in mouse tumor cells. The proposed method succeeds in detecting significant gene functions for tumor-specific T cell dysfunction and reprogramming.

## Methods

### Problem statement

Suppose we have gene expression dataset *X*∈*R*^*m*×*N*^ for *m* genes *g*_1_,⋯,*g*_*m*_ and *N* samples, and also a phenotype *y*∈*R*^*N*^. The gene set *S*={*g*_1_,⋯,*g*_*m*_} is often predefined by a biological pathway or a group of genes with the same gene function. For each gene *i*, a single null hypothesis of interest could be *H*_0*i*_: the *i*th gene is not associated with the phenotype, *i*=1,⋯,*m*. We could calculate *mp*-values {*p*_1_,⋯,*p*_*m*_} by a certain test statistic such as two-sample t-test or Wilcoxon test, which tests *H*_0*i*_ to determine whether the corresponding single gene is significantly associated with the phenotype or not. Our goal is to test an overall null hypothesis *H*_0_: no gene in set *S* is significantly associated with the phenotype, and thus evaluate the association of the whole gene set *S* and the phenotype by calculating a group *p*-value for *S*.

### The methods of FCT, TPM, ARTP and GCP

**Fisher’s combination test (FCT)** [4]

Suppose the *mp*-values are generated from *m* statistical tests based on *m* normally distributed random variables, say, the *m*-th row of *X*, *X*_*m*_. Fisher showed that for independent *p*-values, the statistic

$$\Phi = -2\sum\limits_{i=1}^{m} \ln p_{i} $$

follows a *χ*^2^ distribution with 2*m* degrees of freedom. Based on this theoretical result, a hypothesis testing can be performed to calculate a combined *p*-value. If the original *p*-values are independent, a permutation procedure could be used to empirically estimate the null distribution and thus calculate the combined *p*-value.

**Truncated product method (TPM)** [8]

Truncated product method uses the product of only those *p*-values smaller than a specified threshold *ξ*. The corresponding statistic is defined as

$$W=\prod\limits_{i=1}^{m} p_{i}I(p_{i}\leq \xi), $$

where *I*() is an indicator function, *I*(*p*_*i*_≤*ξ*)=1 if *p*_*i*_≤*ξ* and *I*(*p*_*i*_≤*ξ*)=0 otherwise.

**Adaptive rank truncated product method (ARTP)** [11]

ARTP makes use of multiple candidate truncation integers *K*_1_,⋯,*K*_*L*_ to adaptive combine the the smallest *p*-values. The *m p*-values are first ordered as *p*_(1)_≤*p*_(2)_≤⋯≤*p*_(*m*)_, where *p*_(*k*)_ is the *k*-th smallest *p*-value. A statistic which combines the smallest *K*_*l*_*p*-values are defined by

$$W_{l} = \prod\limits_{i=1}^{K_{l}} p_{(i)}, l= 1,\cdots, L. $$

Let *s*_*l*_ be the *p*-value corresponding to *W*_*l*_, which could be estimated by a permutation procedure. The statistic based on minimum *p*-value can be defined as

$$MinP = \min_{1\leq l\leq L} s_{l}.$$

The adjusted combined *p*-value corresponding to *MinP* is estimated by the permutation procedure.

**Group combined*p*-value (GCP)** [10]

Different from ARTP, which combines the smallest *p*-values using different truncation integers, Group combined *p*-value (GCP) method considers the *p*-values in different specified intervals. Given *J* cutoff values 0<*ξ*_1_<*ξ*_2_<⋯<*ξ*_*J*_<1, GCP defines a statistic

1 $$ GCP = \prod_{j=1}^{J} \left[1-F_{j}\left(\sum\limits_{i=1}^{m}-2 \ln p_{i}I_{\left\{\xi_{j-1} < p_{i} \leq \xi_{j} \right\}}\right)\right]  $$

where *ξ*_0_=0 and *F*_*j*_ is the cumulative distribution function of ${\sum }_{i=1}^{m}-2\ln p_{i}I_{\{\xi _{j-1} < p_{i} \leq \xi _{j} \}}$ for *j*=1,2,⋯,*J*. The permutation procedure is also used to estimate the empirical *p*-value corresponding to GCP.


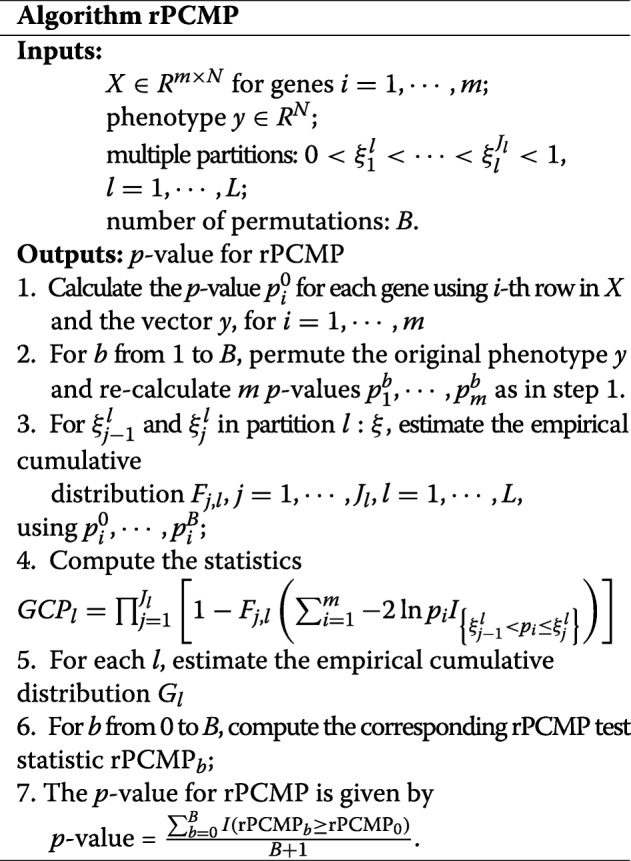


### Combining *p*-values by multiple partitions

In GCP method, it is unclear how to choose cutoff values for calculating the group combined *p*-values. Note that one choice of these cutoffs actually mean a partition of the *mp*-values. Different partitions of these *p*-values will result in different testing results. In this work, we aim to propose a robust method, which accounts for multiple partitions of the *p*-values. Suppose we have *L* partitions of the *mp*-values, say, $0=\xi ^{l}_{0}<\xi ^{l}_{1}<\xi ^{l}_{2}<\xi ^{l}_{3}<\cdots <\xi _{J_{l}}^{l}<1, l =1,\cdots,L$. We borrow the ideas of both ARTP and GCP, and define a rPCMP statistic by integrating the multiple partitions of the *p*-values:

2 $$ {\begin{aligned} \text{rPCMP} \,=\, \prod_{l=1}^{L} \left(1\,-\, G_{l}\left(\prod_{j=1}^{J_{l}} \left[1\,-\,F_{j,l}\left(-2\sum\limits_{i=1}^{m} \ln p_{i}I_{\left\{\xi^{l}_{j-1} < p_{i} \leq \xi^{l}_{j} \right\}}\right)\right]\right)\right), \end{aligned}}  $$

where *F*_*j*,*l*_ is the cumulative distribution function of $-\thinspace 2{\sum }_{i=1}^{m} \ln p_{i}I_{\left \{\xi ^{l}_{j-1} < p_{i} \leq \xi ^{l}_{j}\right \}}$, and *G*_*l*_ is the cumulative distribution function of

3 $$ {GCP}_{l} =\prod_{j=1}^{J_{l}} \left[1-F_{j,l}\left(-2\sum\limits_{i=1}^{m} \ln p_{i}I_{\left\{\xi^{l}_{j-1} < p_{i} \leq \xi^{l}_{j} \right\}}\right)\right].  $$

**Fig. 1:**
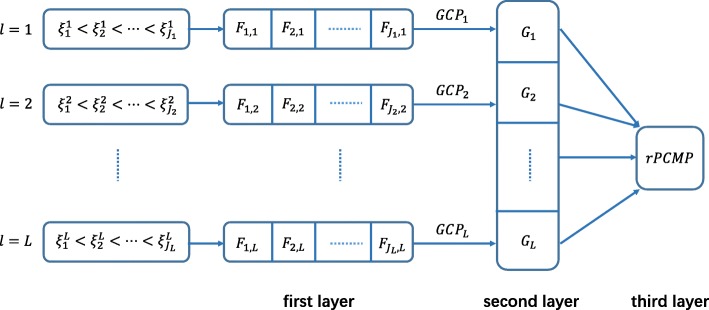
The three-layer hierarchical structure of rPCMP statistic

Note that there are three layers of the rPCMP statistic. The inner-layer distribution *F*_*j*,*l*_ depends on both the partition *l* and the cutoff values $\xi ^{l}_{j-1}$ and $\xi ^{l}_{j}$, the intermediate-layer *G*_*l*_ depends only on the thresholds of the *l*-th partition, and outer-layer statistics integrate all the *L* multiple partitions. ARTP statistic takes two-layer distributions, but both the two layers are different from rPCMP. The three-layer structure of the rPCMP statistic is shown in Fig. 1.

The distribution of the three-layer rPCMP statistic under the overall null hypothesis could be both estimated by generating permutation *p*-values under null distribution. Therefore, to obtain the adjusted *p*-value for the defined statistic rPCMP, we need a three-level permutation procedure [15] with the inner level for estimating *F*_*j*,*l*_, intermediate-layer for estimating *G*_*l*_, and the outer-layer for rPCMP with multiple partitions. However, this would be computationally expensive if *m* is relatively large. Thus a single-layer permutation is used to determine the significance level for rPCMP, which borrows techniques originally designed for gene expression analysis [16]. By this single-layer permutation procedure, we first calculate *p*-values $p^{0}_{1},\cdots,p^{0}_{m}$ for each test on the null hypothesis based on the observed data {*X*,*y*}. We then generate *B* permuted datasets {*X*,*y*_*b*_} by randomly permuting the phenotype *y* to be *y*_*b*_, where 1≤*b*≤*B*, under the null hypothesis. Based on these *B* permuted datasets, we can calculate *p*-values $p^{b}_{1},\cdots,p^{b}_{m}$. By using these *p*-values, we can apply the rPCMP algorithm to obtain the adjusted *p*-values for the rPCMP statistic. The detailed steps are shown in the algorithm rPCMP.

## Results

### Simulation datasets

We generate *N* columns of *X*∈*R*^*m*×*N*^ by multivariate normal distribution with zero mean and a covariance matrix *Σ*∈*R*^*m*×*m*^. For independent case, where genes are assumed to be independent, we just set *Σ* to be an identical matrix. For dependent case, we set *Σ*_*ij*_=*ρ*^|*i*−*j*|^, where the parameter *ρ* is chosen from the set {0,0.1,0.2,0.3}. The number of genes *m* is chosen from {100,200,300,400,500}, and the sample size *N* is chosen as 100. We further generate *y*∈*R*^*N*^ by the following procedure. We first randomly select *T*_1_ rows from *X*, which are assumed as the related genes, and then randomly generate *T*_2_ row vectors by standard normal distribution. *y* is generated by the linear combination of these *T*=*T*_1_+*T*_2_ vectors, with the same coefficient 1/*T*. In our experiments, we fix *T*=30 and vary *T*_1_ from 1 to 30. Note that *T*_1_ is the number of related genes in *X*. The dataset {*X*,*y*} depends on three parameters *ρ*,*m* and *T*_1_.

### Simulation results

For all methods except FCT, there are parameters to be set up. For TPM method, we use 0.5 as the cutoff for all experiments. For ARTP method, the truncation integers are set to be 1 to 10 in all experiments. For GCP, we use the best parameter [0.001, 0.05] suggested by [10]. In our method, 5 groups parameters are used together in all experiments from the sets {[0.01, 0.1], [0.001, 0.05], [0.01, 0.05], [0.001, 0.01, 0.1],[0.001, 0.01, 0.05]}, which are also used in [10].

For each simulation dataset {*X*,*y*}, we first compute *mp*-values for the *m* genes by Student’s t test, and then *B*=1000 permuted *y*s are used to compute *Bp*-values for each gene. We use type-I error and power to measure the performance of the baseline methods and our method. To estimate the type-I error, we set *T*_1_=0, calculate 1000 group *p*-values by randomly generating 1000 *y*s. The type-I error is estimated by the proportion of group *p*-values less than 0.05 among these 1000 values. To estimate the power, we randomly select *T*_1_≥1 rows of *X* for 1000 times and thus can generate 1000 *y*s. With each of these *y*s, we could perform different methods to calculate a group *p*-value. The power for the method could then estimated by the proportion of group *p*-values less than *α*=0.05.

We report the Type I error rates in Table 1 by different methods for *m*∈{100,200,300,400,500} and *ρ*∈{0,0.1,0.2,0.3}. We can see that all methods could obtain very small type I error rates with slight differences.

**Table 1:** Type I error for rPCMP,GCP,ARTP,TPM and FCT

Methods	*m*=100	*m*=200	*m*=300	*m*=400	*m*=500	*m*=100	*m*=200	*m*=300	*m*=400	*m*=500
	*ρ*=0					*ρ*=0.1				
FCT	0.1020	0.0980	0.1110	0.1110	0.1000	0.1210	0.0850	0.0990	0.1170	0.0860
TPM	0.0880	0.0920	0.1010	0.1020	0.1050	0.0980	0.1010	0.1010	0.0920	0.0910
ARTP	0.0770	0.1050	0.0870	0.1160	0.0920	0.1050	0.0980	0.0940	0.0950	0.0910
GCP	0.0880	0.0920	0.0920	0.1130	0.1000	0.0990	0.0930	0.0950	0.0960	0.0940
rPCMP	0.0880	0.1020	0.0800	0.1130	0.0970	0.1020	0.0940	0.0920	0.1010	0.1010
	*ρ*=0.2					*ρ*=0.3				
FCT	0.1300	0.1160	0.1200	0.1070	0.1140	0.1280	0.1460	0.1300	0.1350	0.1210
TPM	0.1060	0.1060	0.1010	0.0830	0.0930	0.0940	0.1010	0.1000	0.1020	0.0930
ARTP	0.1110	0.1030	0.1020	0.0910	0.0950	0.1000	0.1020	0.0930	0.1100	0.0950
GCP	0.1050	0.1030	0.1060	0.0850	0.1020	0.0970	0.0880	0.0830	0.1010	0.0880
rPCMP	0.1070	0.0970	0.0920	0.0880	0.1080	0.0970	0.0970	0.0870	0.1090	0.0930

**Fig. 2:**
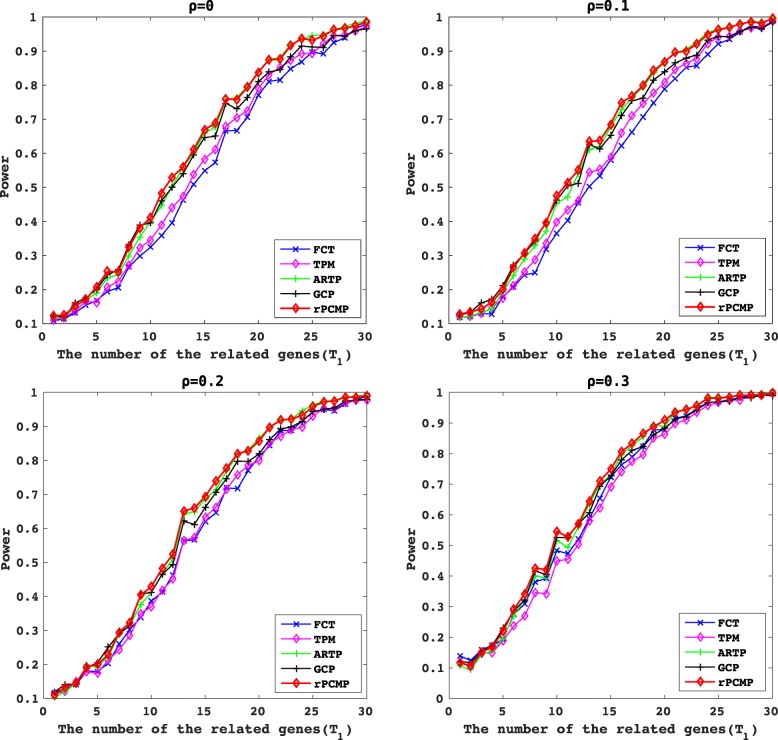
The power of five methods for *T*_1_ genes selected from m=300 genes and varied from 1 to 30

In Fig. 2, we show the change of power with *T*_1_ varied from 1 to 30, for *m*=300 and different choices of *ρ*=0,0.1,0.2,0.3. We can see that the four sub-figures show the advantage of our rPCMP over all other methods. To account for the overall performance for different *T*_1_, we compute further an average area under curve (AAUC) defined as the area under the power curve divided by 30. In Table 2, we report the AAUCs for different *m*∈{100,200,300,400,500} and *ρ*∈{0,0.1,0.2,0.3}. We can see that our method performs the best for almost all the cases.

**Table 2:** Power for rPCMP,GCP,ARTP,TPM and FCT computed by the average area under the curve

Methods	*m*=100	*m*=200	*m*=300	*m*=400	*m*=500	*m*=100	*m*=200	*m*=300	*m*=400	*m*=500
	*ρ*=0					*ρ*=0.1				
FCT	0.7376	0.6198	0.5524	0.5096	0.4603	0.7570	0.6348	0.5749	0.5171	0.4286
TPM	0.7370	0.6376	0.5704	0.5247	0.4701	0.7491	0.6459	0.5927	0.5271	0.4587
ARTP	**0.7642**	0.6726	0.6115	0.5693	0.5190	**0.7729**	0.6823	0.6314	0.5699	0.5072
GCP	0.7363	0.6531	0.6004	0.5641	0.5183	0.7479	0.6646	0.6257	0.5647	0.5085
rPCMP	0.7601	**0.6740**	**0.6174**	**0.5795**	**0.5325**	0.7708	**0.6851**	**0.6411**	**0.5804**	**0.5232**
	*ρ*=0.2					*ρ*=0.3				
FCT	0.7862	0.6789	0.5960	0.5429	0.5210	0.8066	0.7146	0.6487	0.6059	0.5483
TPM	0.7768	0.6735	0.5959	0.5465	0.5186	0.7929	0.6945	0.6297	0.5836	0.5264
ARTP	**0.7978**	0.7035	0.6325	0.5893	0.5567	**0.8093**	0.7209	0.6620	0.6181	0.5670
GCP	0.7797	0.6907	0.6204	0.5881	0.5591	0.7954	0.7094	0.6588	0.6162	0.5661
rPCMP	0.7953	**0.7056**	**0.6373**	**0.6016**	**0.5712**	0.8089	**0.7233**	**0.6721**	**0.6292**	**0.5760**

The best results are marked in blodface

**Fig. 3:**
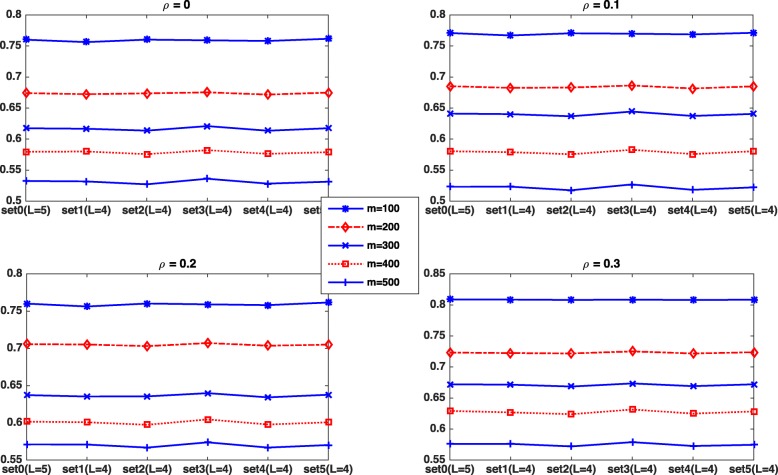
Power for rPCMP computed by the average area under curve with different *ρ*s and cutoff interval sets

To check the robustness of our rPCMP on the number of partitions *L*, we remove each partition set from the partition sets {[0.01, 0.1], [0.001, 0.05], [0.01, 0.05], [0.001, 0.01, 0.1],[0.001, 0.01, 0.05]}(denoted by set0) in turn to generate 5 new parameter setting named set1, set2, set3, set4 and set5. Note that these five parameter settings all have *L*=4 partition sets. In Fig. 3, we show the power of rPCMP computed by average area under curve for each *ρ* with different *m*, where *ρ* varies from the set of {0,0.1,0.2,0.3} and *m* is selected from the set of {100,200,300,400,500}. We can see that our method can perform stably in all cases.

### Demonstration of three-layer statistic of rPCMP

**Fig. 4:**
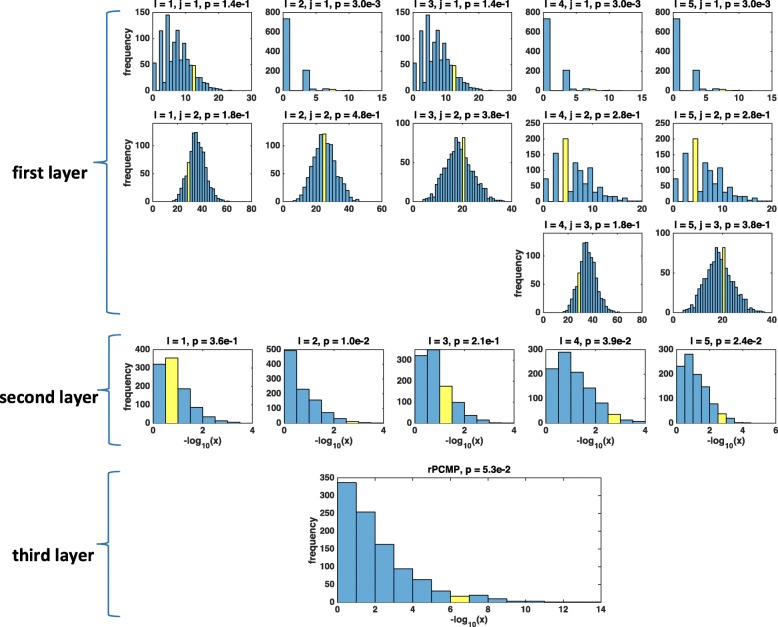
An example of three-layer statistic by a simulation study with *m*=300, *T*=30, *ρ*=0. The top layer shows the empirical distributions of *F*_*j*,*l*_ for the *j*-th group in *l*-th partition.The second-layer shows the empirical distributions of *G*_*l*_, for the *l*-th partition. The third-layer shows the distribution of rPCMP. The observations are marked by yellow in all cases

Figure 4 demonstrates the three-layer structure of rPCMP statistic by a simulation study with *m*=300, *T*=30, *ρ*=0. The top layer shows the empirical distributions of *F*_*j*,*l*_ for the *j*-th group in *l*-th partition. Each *F*_*j*,*l*_ could result in a *p*-value, shown in title of each sub-figure by only choosing the individual *p*-values in the corresponding interval, We can see that these *p*-values are very unstable, and this implies that a statistic combining individual *p*-values in a specified interval is very sensitive to the interval parameters. The second-layer in the figure shows the empirical distribution of *G*_*l*_, for the *l*-th partition. Each *G*_*l*_ actually integrates the information of $\phantom {\dot {i}\!}F_{1,l},\cdots,F_{J_{l},l}$ from the top-layer. Note that the *p*-values obtained by *G*_*l*_ is still unstable, which may have large *p*-values for some *l*s, and small *p*-values for other *l*s. For the third-layer of the figure, rPCMP integrates the optimized information from the second-layer of *G*_*l*_, and thus makes the result stable.

### Applications for identifying related pathways for tumor-specific T cell dysfunction and reprogramming

Dysfunctional tumor-specific CD8 T cells (TST) in solid tumors allow tumors to progress. Immune checkpoint blockade and adoptive T cell therapy has been successfully used in subset of cancer patients, and this shows great potential of TST. However, it is still a problem how to predict which patients will respond to therapy, and it has important implications for cancer immunotherapy to study the epigenetic regulation of T cell dysfunction and therapeutic reprogrammability. Schietinger et al. [17] points out that TST dysfunciton is initially reversible but ultimately becomes irreversible, even after removal of dysfunctional T cells from the tumor. In the study of [18], “Assay for Transposase Accessible Chromatin using Sequence” (ATAC-Seq) [19] was used to assess genome-wide chromatin accessibility changes during T-cell differentiation in tumors compared to acute infection. T cells in mouse tumors are shown in [18] to differentiate through two discrete chromatin states: a plastic dysfunctional state from which T cells can be rescued, and a fixed dysfunctional state in which the cells are resistant to reprogramming. In their study, some membrane proteins such as CD38, CD101, CD30L, CD5, TCF1, IRF4, BCL2, CD44,PD1, LAG3 and CD62L are identified as related to the two chromatin states. In this application, we aim to use ATAC-Seq data to identify related gene functions, which are sets of genes, associated with T-cell dysfunction and reprogramming.

The preprocessed ATAC-seq dataset for mouse is downloaded from the Gene Expression Omnibus with GEO Series accession number GSE89308. Totally 16917 genes are assigned in the ATAC-seq data. The collected 22 mouse samples are labeled using their plastic or fixed dysfunctional chromatin states identified in [18], i.e., they are labeled as 1 for L5 and L7 representing the chromatin remodelling occurred by day 5 and 7, and labeled as 0 for L14,L21,L28,L35 and L60 representing chromatin remodelling occurred by day 14, 21, 28, 35 and 60. We also collect mouse gene ontologies(GO) from http://baderlab.org/GeneSets, and select 2446 GO terms with five to ten genes to perform the analysis.

We apply our rPCMP method to calculate the group *p*-values for all these GO terms, with the same parameters $\xi _{l}^{j}$ as in the simulation study, and permutation time *B*=10,0000. We identify 13 GO terms shown in Table 3 with smallest group *p*-values as the related gene functions to tumor-specific T cell dysfunction and reprogramming. Some of these identified gene functions are related with immune system, including GO:0033007, GO:0002322, GO:0002923, GO:0002921, GO:0002279 and GO:0061081. The gene set GO:0033007 includes immune genes CD300a and CD84, GO:0002322 includes CD180, GO:0002923 includes CD55, GO:0002921 includes CD59b, CD46 and CD59a, GO:0002279 includes CD300a, and GO:0061081 includes CD74,CD36. These genes are highly related to the identified CD family genes in [18]. Besides, another gene set GO0030855 also includes CD family genes CD37. We also found that other gene sets include gene TCF1, which is another name of TCF7, and gene BCL2. These two genes are also identified in [18]. The evidence shows that by our results are well supported by the results in [18], which identifies several CD family genes as related to the chromatin states.

**Table 3:** The identified Gene ontology terms related to chromatin states in mouse T cell

GO terms	GO functions	Genes
GO:0033007	NEGATIVE REGULATION OF MAST CELL ACTIVATION INVOLVED IN IMMUNE RESPONSE	Cd300a,Rabgef1,Hmox1,Cd84,Fer
GO:0002322	B CELL PROLIFERATION INVOLVED IN IMMUNE RESPONSE	Tlr4,Gapt,Cd180,Abl1,Plcl2
GO:0002923	REGULATION OF HUMORAL IMMUNE RESPONSE MEDIATED BY CIRCULATING IMMUNOGLOBULIN	Tnf,Ptprc,Foxj1,Ptpn6,Lta,Susd4,Fcgr2b,Cd55,Nod2
GO:0002921	NEGATIVE REGULATION OF HUMORAL IMMUNE RESPONSE	Foxj1,Ptpn6,Cd59b,Cd46,Susd4,Fcgr2b,Spink5,Cr1l,Cd59a,Serping1
GO:0002279	MAST CELL ACTIVATION INVOLVED IN IMMUNE RESPONSE	Chga,Cd300a,Nr4a3,Milr1,Btk,Ywhaz,Lyn,Snap23,Rasgrp1,Kit
GO:0030885	REGULATION OF MYELOID DENDRITIC CELL ACTIVATION	Havcr2,Flt3l,Klrk1,Tspan32,Il10,Cd37
GO:0036037	CD8-POSITIVE, ALPHA-BETA T CELL ACTIVATION	Ifng,Satb1,Otud5,Tnfsf8,Irf1,Gpr18,H2-T23,Eomes,Bcl2
GO:0061081	POSITIVE REGULATION OF MYELOID LEUKOCYTE CYTOKINE PRODUCTION INVOLVED IN IMMUNE RESPONSE	Gprc5b,Tlr4,Mif,Nr4a3,Cd74,Tlr2,Spon2,Sema7a,Cd36,Fcer1g
GO:1990441	NEGATIVE REGULATION OF TRANSCRIPTION FROM RNA POLYMERASE II PROMOTER IN RESPONSE TO ENDOPLASMIC RETICULUM STRESS	Jun,Nck1,Ppp1r15a,Tmbim6,Nck2
GO:0061525	HINDGUT DEVELOPMENT	Shh,Hoxd13,Gli2,Tcf7,Dact1,Tcf7l2,Ift172
GO:0044336	CANONICAL WNT SIGNALING PATHWAY INVOLVED IN NEGATIVE REGULATION OF APOPTOTIC PROCESS	Ctnnb1,Apc,Wnt1,Tcf7,Mitf
GO:0006582	MELANIN METABOLIC PROCESS	Tyrp1,Mc1r,Dct,Pmel,Myo5a,Vhl,Oca2,a,Cited1,Bcl2
GO:0060442	BRANCHING INVOLVED IN PROSTATE GLAND MORPHOGENESIS	Hoxa13,Shh,Hoxd13,Fgfr2,Esr1,Fem1b,Cd44,Hoxb13,Frs2

To show the better performance of rPCMP than GCP, we also apply GCP to obtain the group *p*-values for all these GO terms with the best cutoff interval suggested by [10]. The results show that GCP could only find five GO terms involving the genes identified by [18], or related to immune systems. These GO terms include GO:0002765, GO:0002826, GO:0002857, GO:0042092 and GO:0002566. Among them, GO:0002826 and GO:0002566 are also discovered by our rPCMP, and only GO:0002857 contains CD family genes while the others don’t have both CD family and BCL2 family genes. This futher shows that rPCMP outperforms GCP on this biological application.

## Discussion

The rPCMP proposed in this work shows robustness and higher statistical power than other existing *p*-value combination methods in most scenarios of the simulation studies. This is highly expected since the compared methods except ARTP only take a partition of *p*-values, while rPCMP takes several partitions into account for combining *p*-values. Although ARTP also takes different truncation points, it neglects the larger individual *p*-values, and thus loses some information. Our rPCMP extends the *p*-value combination technique of GCP by dividing *p*-values to several groups for multiple times, and grouping them first in threshold level and then in partition level. The strategy optimizes the different partitions and and accumulates the advantages from different partitions to improve the power of test significantly, and thus is more robust than GCP with a fixed partition. Simulations studies show that rPCMP outperform other methods in most scenarios, and the applications to ATAC-Seq data further demonstrate its good performance.

The success of rPCMP mostly comes from its three-layer statistical structure, which makes it more flexible to choose the partitions for grouping *p*-values.The hierarchical structure optimizes the inner-level information and transfers it to the outer-level statistics. Similar structure has been used in ARTP, but it loses some statistical power since it has two layers, and removes the large*p*-values using the truncation points. Our method rather keep all the *p*-values in different groups. Although theoretically it may increase the computational load due to the requirement of three layers of permutation procedure, we propose a single-layer permutation procedure to reduce the complexity and shows its effectiveness in experiments. Intuitively, the three-layer structure of the statistic could be generalized to four-layer, by defining different sets of partition sets. However, three-layer structure is good enough in most scenarios, and increasing layer will introduce more complicated parameter sets and computational complexity.

In our experiments, the multiple partitions are defined by five popular sets of thresholds: [0.01, 0.1],[0.001,0.05], [0.01, 0.05], [0.001, 0.01, 0.1],[0.001, 0.01, 0.05]. Note that the thresholds are all very popular cutoffs used in statistical field and these sets are also used in [10]. We suggest to use these partition sets in the applications. In prin76ciple, a large value of permutation time *B* is preferred in the experiments because it can yield more accurate null distribution and thus obtain more accurate *p*-value. However, a very large *B* results in extensive computational load. Thus in our experiments, we use *B*=1000 in simulation studies and *B*=10,000 in applications, to balance the tradeoff.

## Conclusion

We propose a robust statistical method rPCMP by using multiple partitions of *p*-values in this work, to reduce the sensitivity of GCP method. The rPCMP statistic is a three-layer statistic, which takes into consideration the different partitions of the individual *p*-values. This three-layer statistic could be empirically estimated by a single-layer permutation procedure. Type I error rates and statistical power are used to evaluate our rPCMP method. The simulations studies show that our proposed rPCAMP test method perform more powerful than some existing *p*-value combination methods, with low type I error rates. Our method is finally applied to a ACTC-Seq dataset, to find the related gene functions for chromatin states in mouse tumor cells. The proposed method succeeds in detecting significant gene functions for tumor-specific T cell dysfunction and reprogramming. One future research could be to further adapt the current rPCMP for highly correlated individual genes.

## Abbreviations

ARTP: Adaptive rank truncated product method
ATAC-Seq: Assay for transposase accessible chromatin using sequence
FCT: Fisher’s combination test
GCP: Group combined *p*-value
rPCMP: robust *p*-value combination by multiple partitions
SNPs: Single nucleotide polymorphisms
TPM: Truncated product method

## References

[1] WTCC C (2007). Genome-wide association study of 14,000 cases of seven common diseases and3,000 shared controls. Nature.

[2] Hunter DJ, Kraft P, Jacobs KB, Cox DG, Yeager M, Hankinson SE (2007). A genome-wide association study identifies alleles in FGFR2 associated with risk of sporadic postmenopausal breast cancer. Nat Genet.

[3] Subramanian A, Tamayo P, Mootha VK, Mukherjee S, Ebert BL, Gillette MA (2005). Gene set enrichment analysis: A knowledge-based approach for interpreting genome-wide expression profiles. Proc Natl Acad Sci.

[4] Fisher RA (2017). Statistical methods for research workers.

[5] Zheng G, Wu CO, Kwak M, Jiang W, Joo J, Lima JA (2012). Joint analysis of binary and quantitative traits with data sharing and outcome-dependent sampling. Genet Epidemiol.

[6] Li Q, Hu J, Ding J, Zheng G (2014). Fisher’s method of combining dependent statistics using generalizations of the gamma distribution with applications to genetic pleiotropic associations. Biostatistics.

[7] Hess A, Iyer H (2007). Fisher’s combined p-value for detecting differentially expressed genes using Affymetrix expression arrays. Bmc Genomics.

[8] Zaykin DV, Zhivotovsky LA, Westfall PH, Weir BS (2002). Truncated product method for combining *P*-values. Genet Epidemiol.

[9] Dudbridge F, Koeleman BPC (2003). Rank truncated product of *P* -values, with application to genomewide association scans. Genet Epidemiol.

[10] Hu X, Zhang W, Zhang S, Ma S, Li Q (2011). Group-combined *P*-values with applications to genetic association studies. Bioinformatics.

[11] Yu K1, Li Q, Bergen AW, Pfeiffer RM, Rosenberg PS, Caporaso N (2009). Pathway analysis by adaptive combination of P-values. Genet Epidemiol.

[12] Taylor J, Tibshirani R (2006). A tail strength measure for assessing the overall univariate significance in a dataset. Biostatistics.

[13] Jiang B, Zhang X, Zuo Y, Kang G (2011). A powerful truncated tail strength method for testing multiple null hypotheses in one dataset. J Theor Biol.

[14] Chen H, Pfeiffer RM, Zhang S (2013). A Powerful Method for Combining - Values in Genomic Studies. Genet Epidemiol.

[15] Westfall P, Young B (1993). Resampling-Based Multiple Testing: Examples and Methods for *P*-Value Adjustment.

[16] Ge Y, Dudoit S, Speed T (2003). Resampling-based multiple testing for microarray data analysis. Test.

[17] Schietinger A, Philip M, Krisnawan VE, Chiu EY, Delrow JJ, Basom RS (2016). Tumor-Specific T Cell Dysfunction Is a Dynamic Antigen-Driven Differentiation Program Initiated Early during Tumorigenesis. Immunity.

[18] Philip M, Fairchild L, Sun L, Horste EL, Camara S, Shakiba M (2017). Chromatin states define tumour-specific T cell dysfunction and reprogramming. Nature.

[19] Buenrostro JD, Giresi PG, Zaba LC, Chang HY, Greenleaf WJ (2013). Transposition of native chromatin for fast and sensitive epigenomic profiling of open chromatin, DNA-binding proteins and nucleosome position. Nat Methods.

